# Effects of elevational range shift on the morphology and physiology of a carabid beetle invading the sub-Antarctic Kerguelen Islands

**DOI:** 10.1038/s41598-020-57868-0

**Published:** 2020-01-27

**Authors:** T. Ouisse, E. Day, L. Laville, F. Hendrickx, P. Convey, D. Renault

**Affiliations:** 1 Université de Rennes 1, UMR CNRS 6553 Ecobio, 263 Avenue du Gal Leclerc, 35042 Rennes, cedex France; 2 Ghent University, Department of Biology, terrestrial ecology unit, K.L. Ledeganckstraat 35, B-9000 Ghent, Belgium; 3 Royal Belgian Institute of Natural Sciences, Vautierstraat 29, B-1000 Brussels, Belgium; 4 British Antarctic Survey, Natural Environment Research Council, High Cross, Madingley Road, Cambridge, CB3 0ET United Kingdom; 5 Institut Universitaire de France, 1 Rue Descartes, 75231 Paris, cedex 05 France

**Keywords:** Behavioural ecology, Climate-change ecology, Ecophysiology, Invasive species, Population dynamics, Ecology

## Abstract

Climatic changes can induce geographic expansion and altitudinal shifts in the distribution of invasive species by offering more thermally suitable habitats. At the remote sub-Antarctic Kerguelen Islands, the predatory insect *Merizodus soledadinus* (Coleoptera: Carabidae), introduced in 1913, rapidly invaded coastal habitats. More recent colonisation of higher elevation habitats by this species could be underlain by their increased thermal suitability as the area has warmed. This study compared the effect of elevational range shift on the morphology and physiology of adult *M. soledadinus* sampled along two altitudinal transects (from the foreshore to 250 m a.s.l.) and a horizontal lowland transect orthogonal to the seashore (400 m length). Although high inter-individual and inter-transect variations in the traits examined were present, we observed that body mass of males and females tended to decrease with elevation, and that triglyceride contents decreased with distance from the shore. Moreover, protein contents of females as well as those of 26 metabolites were influenced significantly by distance to the foreshore. These results suggest that future climate change at the Kerguelen Islands will further assist the colonisation of lowland inland and higher altitude habitats by this aggressively invasive predator, by making previously sub-optimal habitats progressively more suitable.

## Introduction

The geographic distributions and abundances of ectothermic groups such as insects are shaped in part by climatic conditions. In the current context of rapid global and regional climatic changes, altitudinal and latitudinal range shifts have been documented for various ectothermic taxa^1–3^. Moreover, increasing air temperature may favour human-assisted translocation and establishment of alien insects into new geographic areas^4,5^. Climate change can increase both survival and functional capabilities of introduced insects^6^, and may lower or remove barriers to invasion^1,4,7–9^. Changing climatic conditions can also facilitate the establishment of alien insects indirectly by altering community assemblages^10–13^ and increasing niche availability^14^.

In parts of the polar regions, contemporary climatic changes are occurring more rapidly than elsewhere, and greatly modify climate envelopes from micro- to macro-geographical scales^15–17^. These changes are opening opportunities for newly arrived species to successfully establish^10^, and in some cases become invasive^10,18–20^. The sub-Antarctic Kerguelen archipelago is no exception, with mean air temperatures that have increased by 1.7 °C over the period 1951–2010, while precipitation has decreased drastically since the 1990s^20^. The establishment and geographic spread of aphids, the blowfly *Calliphora vicina* and the carabid beetle *Merizodus soledadinus* on the archipelago have likely been assisted by these changing conditions^20,21^.

The predaceous *M. soledadinus* was introduced from the Falkland Islands to the Kerguelen archipelago in 1913^22,23^. In the archipelago, this insect preferentially feeds on fly larvae^24^, and has year-round activity^25^. The main habitat conditions within the natural distribution of *M. soledadinus* (Falkland Islands, Patagonia) overlap at least partly with the climatic conditions at the Kerguelen Islands^18,26^, which suggests that this wingless beetle was likely physiologically pre-adapted to the chronically cool oceanic climate of the archipelago. Adult beetles perform well at cool temperatures, although the studies of Lalouette *et al*.^27^ and Laparie and Renault^21^ both suggest that the insects are currently in the lower part of their thermal comfort zone at the Kerguelen Islands.

Long-term monitoring of the distribution of *M. soledadinus* in the Kerguelen archipelago revealed that its geographic distribution and population expanded rapidly in the early 1990s^20,24^. The beetle now has a large range, occupying many coastal areas and expanding along hydrographic networks^28^, where trophic resources and water are available. Recently, populations of *M. soledadinus* have colonised habitats at higher altitude^29^, with individuals now being found up to 400 m above sea level (a.s.l.), where environmental conditions were previously assumed to be unfavourable. Nowadays, mean annual temperature at 400 m a.s.l. ranges between 3 °C and 4 °C^30^, with a temperature lapse rate of 0.6–0.7 °C per 100 m increase in altitude typical in alpine regions and at the sub-Antarctic islands^30–32^. Thus, the mean annual temperature at 400 m a.s.l. is likely to have ranged between 0.3 °C and 2.3 °C in the 1950s at the Kerguelen Islands. Even though arthropod diversity generally progressively declines with increasing altitude, as reported for mites and springtails on Marion Island^33^ and for aphids at the Kerguelen Islands^34^, the distribution of the potential arthropod prey of *M. soledadinus* should also be altered with climate warming along altitudinal gradients^35^.

The recent altitudinal expansion of *M. soledadinus* in the archipelago may be the result of progressively more suitable thermal conditions being experienced at higher altitudes, which may in addition be associated with potential prey (introduced and native Diptera larvae, annelids, micro-arthropods) expanding their ranges toward higher altitudes. If the geographic expansion of *M. soledadinus* towards higher altitude habitats is primarily driven by the movement of a suitable climatic envelope, i.e. the progressive colonisation of suboptimal altitudinal habitats, we would expect to observe the accumulation of cold-stress molecular markers in this insect. In this work, we thus compared the physiological characteristics of adult *M. soledadinus* along altitudinal invasion gradients, by sampling beetles along two replicated transects from the foreshore (0 m a.s.l.) to 250 m a.s.l. An additional horizontal transect (400 m horizontal transect orthogonal to the seashore) was completed to take into account the general reduction in the abundance and diversity of trophic resources with increasing distance from the seashore, and possible impacts on the morphology and physiology (body size, body composition, metabolic phenotypes) of the adult beetles. For all sampled insects, morphological traits and total concentrations of three general reserve compounds in insects (proteins, triglycerides and glycogen) were measured. Metabolic phenotypes of *M. soledadinus* adults were compared among insects sampled at known altitude and/or proximity to the coast *via* Gas Chromatography – Mass Spectrometry analyses (GC-MS). As we hypothesised that range expansion reflects recent climate change, we predicted that higher altitudes should be associated with an increase of adult body size (temperature-size rule). In parallel, we also predicted differentiation of metabolic phenotypes between lowland and highland beetles, in particular for compounds involved in glycolysis and the tricarboxylic acid cycle that are likely to be affected by habitat temperature, and the accumulation of cold stress markers in the form of polyols or free amino acids.

## Results

### Morphological changes along the transects

**Figure 1:**
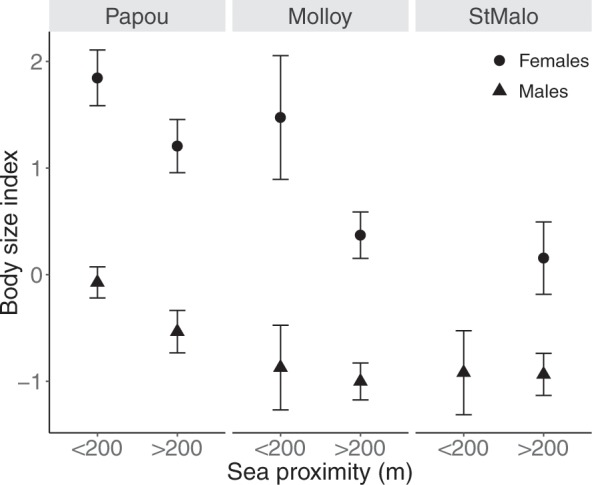
Variations in the body size (mean ± Standard Error [SE]) measured in male and female *M. soledadinus* sampled from the coast to the inland end of each transect (Papous: horizontal transect; Molloy and St. Malo: altitudinal transects). The computed body size value corresponds to the coordinates of the projection of the morphological measurements of each individual on the first axis of a Principal Component Analysis.

Males of *M. soledadinus* were characterised by smaller body size than females (F = 109.77, df = 1, *P* < 0.001, N_Males_ = 287, N_Females_ = 196, see Fig. 1 and Supplementary Table S1 and S2), exemplified by the elytra length of females measuring 3.44 ± 0.17 mm (mean ± SD), while those of males were 3.29 ± 0.16 mm. Body size was not significantly influenced by altitude (F = 0.019, df = 1, *P* = 0.89, N_Males_ = 149, N_Females_ = 117), despite body mass of both males and females decreasing with altitude (Supplementary Table S1 and S2). Individuals sampled closer to the shore had larger body sizes than those sampled further inland (F = 8.34, df = 1, *P* = 0.004, N = 485). A positive but marginally non-significant effect of longitude (F = 7.14, df = 1, *P* = 0.07, N_StMalo_ = 114, N_Molloy_ = 152, N_AnsePapous_ = 219) highlighted possible inter-site differences, with an eastward increase in body sizes of individuals (St. Malo < Molloy < Anse des Papous).

### Variations in glycogen, protein and triglyceride contents

The glycogen contents of the samples were not affected by any of our explanatory variables (Altitude: F = 0.59, df = 1, *P* = 0.44, N = 158; Coast proximity: F = 2.01, df = 1, *P* = 0.16, N = 238; Sex: F = 2.78, *P* = 0.097, N_Males_ = 130, N_Females_ = 108).

**Figure 2:**
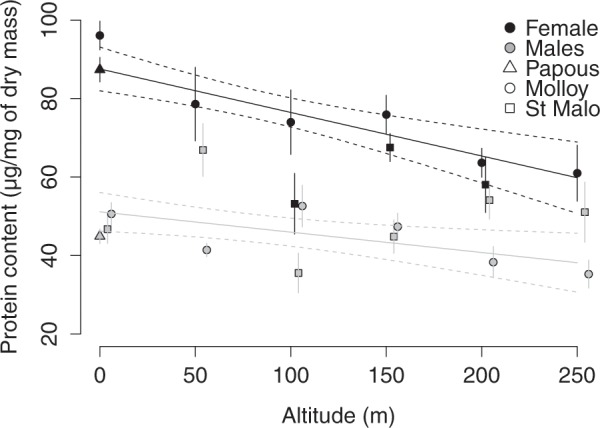
Linear regressions between the variations in protein contents measured in male and female *M. soledadinus* and altitude in the three transects (Papous: horizontal transect; Molloy and St. Malo: altitudinal transects; *dots*: mean ± SE; *solid lines*: regression curves; *dotted lines*: 95% CI). The statistical analyses (ANOVA type II) reported a significant effect of altitude on the protein contents in both males (F = 8.46, df = 1, P < 0.001, N = 90) and females (F = 10.57, df = 1, P < 0.001, N = 68).

Females had significantly higher amounts of proteins than males (F =205.19, df = 1, *P* < 0.001, N_Males_ = 130, N_Females_ = 108, see Fig. 2). Individuals of both sexes sampled in proximity to the shore had higher amounts of proteins (F = 4.14; df = 1, *P* = 0.04, N = 238). The significant interaction between altitude and sex (F = 4.33, df = 1, *P* = 0.04, N = 238) highlighted distinct responses depending on the sex, as female protein content decreased with increasing altitude, while protein content remained stable in males. A significant interaction between sex and longitude (F = 12.78, df = 1, *P* < 0.001, N = 238) again highlighted possible site-specific differences between the two sexes: at the transects completed at Anse des Papous and Molloy, protein amounts were very distinct between males and females, while at St. Malo the values were more similar. However, this difference might also be an artefact of the reduced number of sampling sites from which females were obtained in the St. Malo transect.

**Figure 3:**
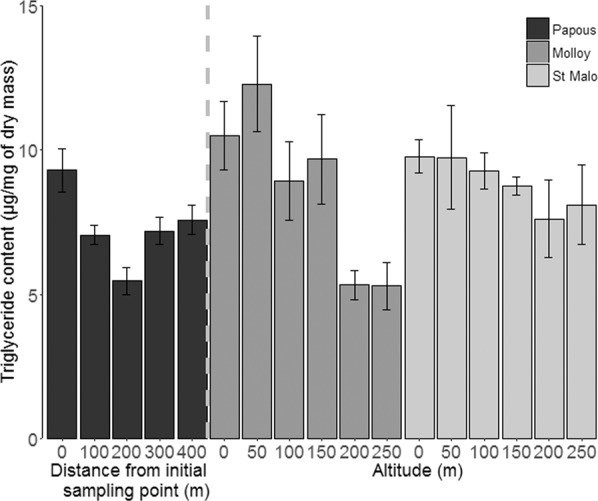
Variations in triglyceride contents (mean ± SE) measure in adult *M. soledadinus* as a function of the distance from the initial sampling point (horizontal transect of Anse des Papous) or as a function of sampling altitude (altitudinal transects of Molloy and St. Malo).

Finally, proximity to the coast had no effect on triglyceride content (F = 2.35, df = 1, *P* = 0.13, N = 100), while triglyceride content co-varied negatively with altitude (F = 19.74, df = 1, *P* < 0.001, N = 70, see Fig. 3). The significant effect of longitude (F = 19.26, df = 1, *P* < 0.001, N = 100) again highlighted site-specific responses, as individuals collected from the two altitudinal transects had higher amounts of triglycerides than those from the horizontal transect.

### Metabolic fingerprinting

**Figure 4:**
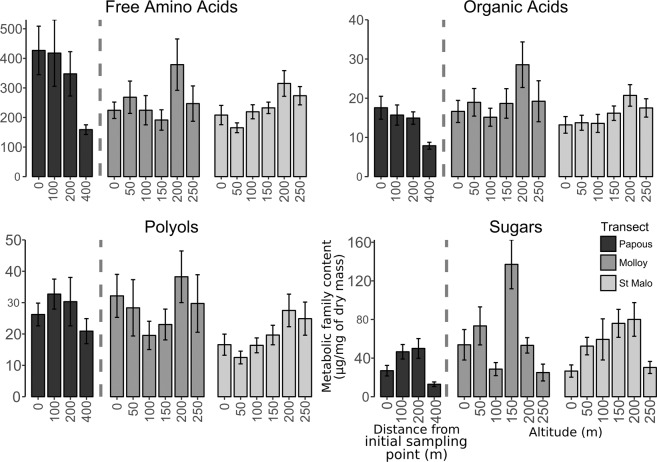
Content variations (mean ± SE) in the five metabolite families investigated along the three transects (from darker to lighter: the horizontal transect of Anse des Papous [0, 100, 200 and 400 m from the initial sampling point], and the altitudinal transect of Molloy and that of St. Malo [0, 50, 100, 150, 200 and 250 m a.s.l. for both transects]). Contents of metabolites belonging to each family were summed and expressed as µg.mg^−1^ dry mass. Top left and right figures of the panel: free amino acids and organic acids, respectively; bottom left and right figures of the panel: polyols and sugars, respectively.

The total amounts of metabolites of three metabolic families (free amino acids, organic acids and polyols) were significantly influenced by altitude. Beetles sampled at 0, 200 and 250 m a.s.l. had higher amounts of free amino acids (F = 4.04, df = 1, *P* = 0.048, N = 59) and polyols (F = 6.64, df = 1, *P* = 0.012, N = 59) than found in beetles sampled at the intermediate points at 50, 100 and 150 m a.s.l. (Fig. 4). The amounts of organic acids were positively correlated with altitude (F = 12.49, df = 1, *P* < 0.001, N = 59), with a regular increase from 150 m upwards in the beetles sampled along the St. Malo transect, and concentration peaking in beetles from the Molloy transect sampled at 200 m a.s.l.

Samples collected near the coast had increased amounts of total free amino acids relative to those collected further inland (F = 5.89, df = 1, *P* = 0.017, N = 91); this difference was strongly influenced by the lower amounts measured in the beetles sampled at 400 m inland along the horizontal Papous transect. Longitude effects on metabolic family contents were also found, as beetles sampled along the horizontal transect had higher amounts of free amino acids (F = 6.29, df = 1, *P* = 0.014, N = 91) compared with the altitudinal transects, and beetles from the St. Malo transect had lower amounts of polyols (F = 11.21, df = 1, *P* = 0.0012, N = 91) compared to the other transects. None of our explanatory variables had significant effects on the amount of sugars (coast proximity: F = 1.81, df = 1, *P* = 0.18, N = 91; altitude: F = 0.03, df = 1, *P* = 0.86, N = 91; longitude: F = 0.23, df = 1, *P* = 0.63, N = 91).

**Figure 5:**
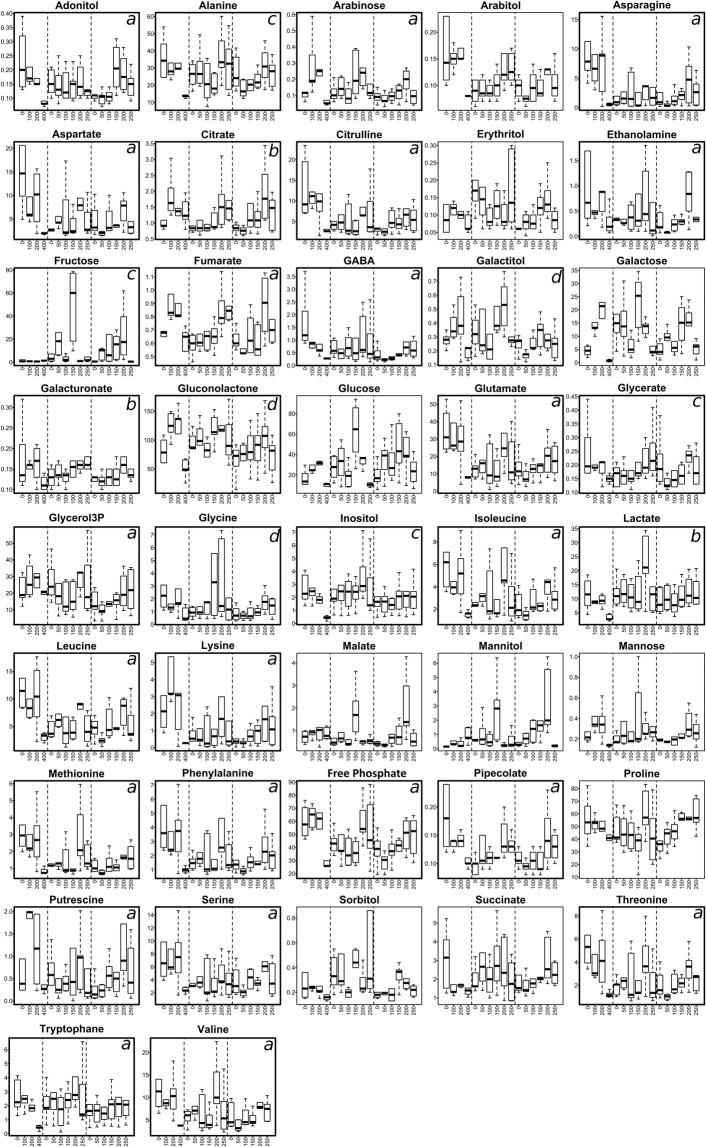
Changes in concentration (µg.mg^−1^ dry mass) of the 42 metabolites quantified observed along the sampling transects. The dotted lines separate the three transects of Anse des Papous (0, 100, 200 and 400 from the initial sampling point), Molloy (0, 50, 100, 150, 200 and 250 m a.s.l.) and St. Malo (0, 50, 100, 150, 200, 250 m a.s.l.), respectively. Metabolites showing significant variations are bold-framed: (**a**) combined effect of coast proximity and longitude, (**b**) altitude effect, (**c**) coast proximity effect, (**d**) longitude effect (inter-site differences). Results of the corresponding statistical tests are presented in the Supplementary Table S3.

The MANOVA performed on the matrix of 42 compounds revealed significant effects of altitude (approx. F_42,45_ = 2.87, *P* < 0.001, N = 91), coast proximity (approx. F_42,45_ = 2.61, *P* < 0.001, N = 91) and the interactions between coast proximity and longitude (F_42,45_ = 2.6, *P* < 0.001, N = 91) on individual metabolite concentrations. Changes in the concentrations of the 42 detected and quantified metabolites along each transect are presented in Fig. 5. The amounts of 26 metabolites were influenced by the sampling distance to the foreshore (Supplementary Table S3). Fourteen metabolites, including 10 free amino acids (Supplementary Table S3), were significantly influenced by both coast proximity and longitude. This result was mainly due to low levels of GABA, glutamate, lysine and threonine quantified from the beetles from the Anse des Papous transect sampled at 400 m inland (Fig. 5, Supplementary Table S3). Conversely, beetles sampled at 200 and 250 m a.s.l. along the altitudinal transects displayed high levels of several metabolites (Supplementary Table S3). Three metabolites (citrate, lactate and galacturonate) co-varied positively with altitude. Inter-site variations were found, with decreased levels of arabinose, galactitol and gluconolactone in samples from the St. Malo transect, while beetles sampled along the Anse des Papous transect had increased levels of fructose, glycerol-3-phosphate (glycerol3P), pipecolate, putrescine and serine compared to the altitudinal transects (Supplementary Table S3).

**Figure 6:**
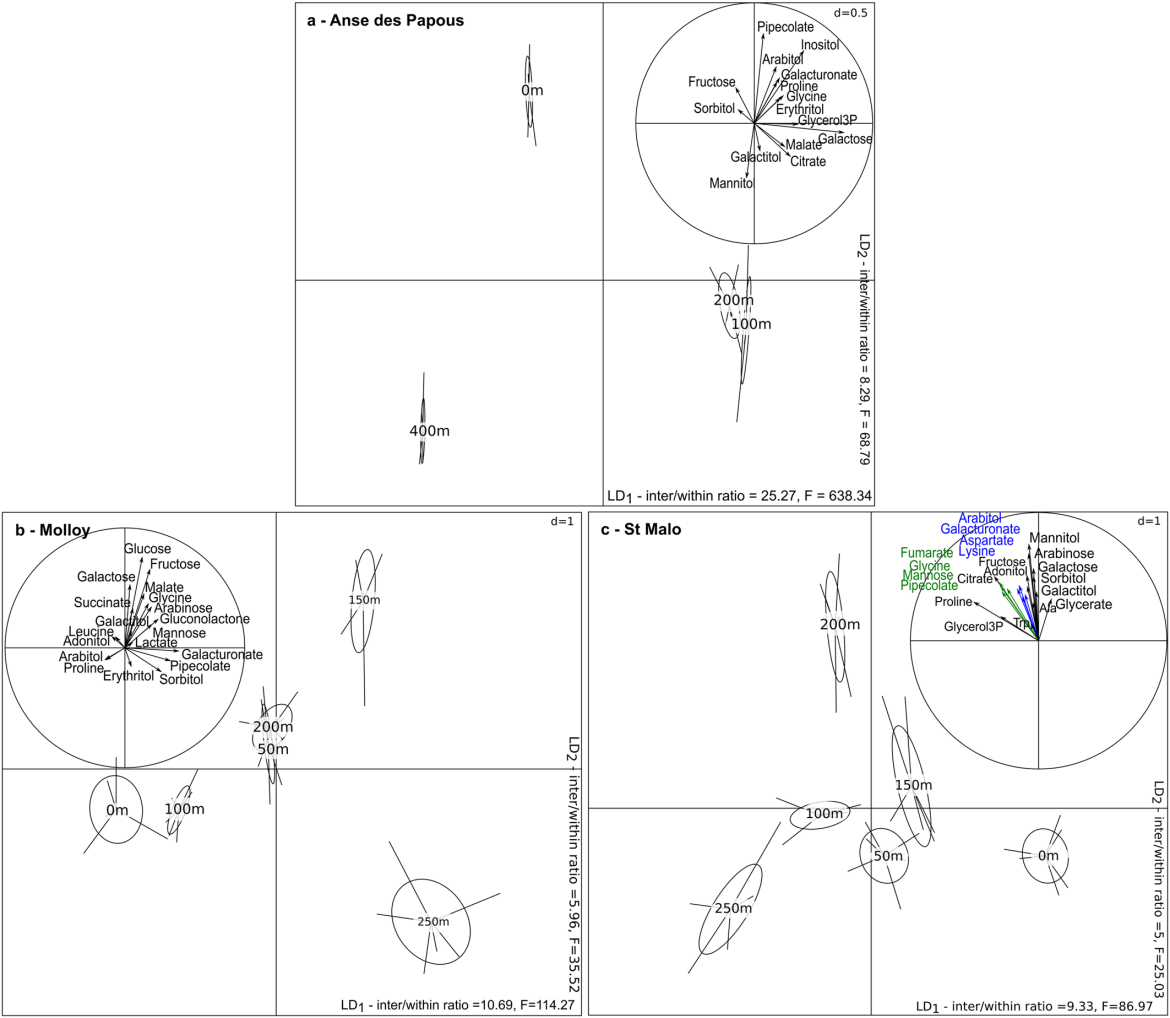
Projection of the metabolic profiles of adult *M. soledadinus* sampled along each of the three transects onto the first two axes of the LDA. Each sampling site is characterised by its distance from the initial sampling point (for the horizontal transect of Anse des Papous – (**a**)) or by its altitude (Molloy and St. Malo altitudinal transects – (**b**,**c**)). The correlation circles depict the relationship between each metabolite retained in the analyses (compounds sharing less than 80% correlation) and the discriminant axes. For each axis, the ratio of inter-class and within-class inertias, and the associated F-statistics are provided.

Metabolic variations were further examined for each transect separately in order to assess if similar patterns could be observed among transects when the sampling groups were discriminated with LDA. For Anse des Papous, the first axis of the LDA (37.2% of the total variance) separated the samples collected at 0 and 400 m inland from those collected at 100 and 200 m inland (Fig. 6a). This discrimination was partly explained by higher amounts of galactose and glycerol-3-phosphate in the two latter insect groups. The second axis (34.7% of the total variance) sorted the groups according to the distance from the initial sampling point. This axis was mainly supported by the co-varying metabolites pipecolate and succinate (only pipecolate is represented on the LDA, as these two compounds have >80% correlation), whose concentrations were increased in groups closest to the coast. Conversely, higher mannitol concentrations were measured in groups sampled furthest from the coast.

For the altitudinal transect conducted at Molloy, the first axis of the LDA accounted for 27% of the total variance and separated the 150 and 250 m a.s.l. sampling points (Fig. 6b). Insects sampled at 150 m a.s.l. had higher amounts of galactose, fructose, glycine and malate. On the second axis (24.5% of the total variance), beetles from groups sampled at 0 and 100 m a.s.l. were separated from those sampled at 150 and 250 m a.s.l., the latter being characterized by increasing concentrations of galacturonate and pipecolate. On both axes, the groups sampled at 50 m and 200 m a.s.l. overlapped.

In the second altitudinal transect (St. Malo, Fig. 6c), the first axis of the LDA (26.2% of the total variance) generally separated the sampling groups along the altitudinal gradient. The second axis (22.6% of the total variance) discriminated the insects sampled at 200 m a.s.l. from the other sampling points, due to increased amounts of arabinose, galactose, mannitol and sorbitol in the former group.

## Discussion

The carabid beetle *M. soledadinus* is a successful invasive species at the Kerguelen Islands^20,28^. Its geographic distribution was restricted to coastal areas for many decades after its introduction^29^ but, since the early 2000s, it has been recorded increasingly frequently at higher altitudes. In the present study, we hypothesised that the ongoing colonisation of altitudinal habitats over the past decade results from increased habitat suitability due to altered thermal conditions through the climatic changes occurring in the archipelago^20^.

Both coast proximity (used to differentiate the higher amount of trophic resources available in the first 200 m away from the seashore, compared to further inland) and altitude had effects on the morphology and physiology of adult *M. soledadinus*, revealing an overall lower quality of individuals sampled further from the coast and at altitude. Even though significant climate warming has occurred since the 1950s at the Kerguelen Islands, this does not alter the altitudinal temperature decrease of 0.6–0.7 °C for each increase of 100 m in elevation in the sub-Antarctic islands^30,32^, with the rate of thermal decrease being the greatest from 0 to 150 m a.s.l.^36^. Temperature is therefore expected to decrease by 2.4 to 2.8 °C between sea level and the highest sampling point in our experimental design. As mean monthly soil temperatures are less than 3.8 °C for 6 months of the year, and less than 2.4 °C for 4 months in lowland habitats^27^, specimens of *M. soledadinus* sampled above 150 m a.s.l. must spend a significant part of the year at temperatures around 0–1 °C. These thermal conditions are restricting for an insect whose acute thermal sensitivity in this thermal range has been reported previously: metabolic activity and efficiency of adult *M. soledadinus* are considerably reduced when temperature falls to around 4 °C^27^, and thermal conditions become increasingly suboptimal for adult *M. soledadinus* as temperatures approach 0 °C^21,27^. Despite ongoing climatic changes, and as expected, it is therefore very likely that adult *M. soledadinus* at higher levels in the two altitudinal gradients still experience significant temperature-imposed resource and physiological limitations.

Triglyceride contents were overall higher in the two altitudinal transects compared to the horizontal one. This finding could indicate energetic adjustments according to the nature of prey available in horizontal *versus* altitudinal transects. Consistent with this surmise, diet-related differences have previously been measured in the body composition of adult *M. soledadinus*^21^.

Triglyceride amounts of both sexes, and protein amounts of females, decreased with altitude. In a previous study, we did not observe variations in the total number of eggs carried by female *M. soledadinus* over the year^25^, and they may be capable of multiple egg maturation cycles. At the Kerguelen Islands, trophic resources become more patchy and sporadic at higher altitudes, limiting nutrient availability for the voracious adults of *M. soledadinus*. In females, the reduction of available resources may explain the lower amounts of triglycerides and proteins, as the production of vitellogenin and other yolk- and egg-related compounds are energy-demanding. In parallel, the structure and abundance of vegetation changed along the two altitudinal transects: from grassland dominated by *Poa annua* (itself a non-native species) at sea level, through continuous cover of *Acaena magellanica* at mid-levels, and progressively becoming scarcer and patchy in rocky fellfields at 250 m a.s.l. (Day E., Laville L., pers. obs). Changes in vegetation cover are known to impact the structure and diversity of invertebrate communities^37^, in turn potentially affecting trophic resources qualitatively and quantitatively for both larvae and adults of *M. soledadinus*. As thermal and trophic resources are more restricting with increasing altitude, resource limitation may have progressively reduced the capacity for energy storage, a finding that is also consistent with the reduction of body mass of the insect along the elevation gradient.

A range of studies have reported decreases in development rates in insects of similar size when energy assimilation is restricted (e.g.^38–40^, and this may result in a progressive reduction in body size along altitudinal gradients^41,42^. Larger insects may require a prolonged developmental time, which may be disadvantageous for insects thriving at higher elevations who experience shorter active seasons, and more particularly for the non-diapausing species that cannot develop over multiple years^43^. At the sub-Antarctic Heard Island, Chown and Klok^2^ observed that body size of weevils decreased with altitude, but this finding contrasted with the pattern reported for these insects from Marion Island. In our study, we did not observe changes in the body size of adult *M. soledadinus* sampled along the altitudinal gradients, while body mass progressively reduced. Where resource limitation occurs, either resulting from thermally-induced constraints or lower availability of trophic resources, the rate of growth of juvenile insects, which is partly driven by protein synthesis^44^, should proceed more slowly. As the body size of *M. soledadinus* from the altitudinal gradients was similar to those from the lowland transect, this suggests that the duration of development may be extended in altitudinal habitats.

Several essential amino acids, including isoleucine, leucine, methionine, phenylalanine, tryptophan, threonine and valine, were present in lower amounts in the beetles as distance to the coast increased (>400 m for the horizontal transect, most often from >50 m for the altitudinal transects). Nutrient limitation in essential amino acids can greatly constrain fecundity^45^, as these metabolites contribute a significant proportion of egg carbon content. Our data may suggest a higher food intake by adult *M. soledadinus* from habitats near the coast, an assumption which is consistent with the variation in protein concentration which reduced as the distance to the coast increased. Furthermore, amino acids represent a key source of nitrogen for insects, and nutrient limitation during the larval stage can have significant consequences on the phenotypic traits of the adult, by modifying developmental rates, which in turn affects fitness^46^. Indeed, several proteins of larval origin are further catabolised by female insects during egg production^45,47^, while additional latent effects of nutrient limitation during larval stages can also be expected, affecting adult immune function, reserve compound storage, or increasing oxidative stress^46^.

The overall inter-site metabolomic comparison revealed that insects from the three transects were characterised by distinct metabolic phenotypes, despite the relatively small geographic distances separating them. Metabolic phenotypes of the specimens from the two altitudinal transects (Molloy and St. Malo) did not overlap, further suggesting that insects experienced distinct ecological conditions along these two transects. Beetles sampled from the St. Malo transect were characterised by higher amounts of polyols and of four metabolites including two amino acids (GABA and glycine) compared to the two other transects. Thermal conditions may be more limiting in this site, St. Malo being closer to the western part of the archipelago, where the climate is colder and wetter. The progressive increase in amounts of polyols is consistent with this hypothesis, as these compounds are typically involved in the physiology of cold tolerance in insects^48^. St. Malo is also the site *M. soledadinus* colonised earliest of those examined in this study (known to have been present since 1996^24^). This longer period of local presence may have had a greater impact on the prey community, by altering qualitatively and quantitatively the available trophic resources.

Metabolic phenotypes of adult *M. soledadinus* from the horizontal transect (Anse des Papous) strongly differed between the insects collected at 0 and 100 m (the latter grouped with the metabolic phenotypes of adult *M. soledadinus* sampled at 200 m), and from those sampled 400 m from the coast. The insects obtained close to the coast (0 m) were characterised by high amounts of the metabolites inositol and pipecolate. At this sampling site, carabid beetles can thrive in concentrations of marine debris where salinity ranges from 35 to 70 parts per thousand, thus imposing osmoregulatory constraints to individuals. Insects exposed to saline conditions often accumulate specific osmolytes^49^, including inositol or pipecolate, as previously reported by Hidalgo *et al*.^50^ in adult *M. soledadinus* experimentally subjected to increasingly saline conditions. When sampled 400 m from the seashore, beetles exhibited lower concentrations of amino acids, tricarboxylic cycle intermediates and sugars as compared with those from the three other groups. Even in the absence of data on the metabolic rates of these specimens, this result may be explained by a reduced activity of energetic metabolism resulting from the lower availability of trophic resources. Consistently, the concentrations of three (free amino acids, intermediate acidic metabolites and amines) out of the four defined metabolic families in insects from the 400 m sample from the horizontal transect were similar to the pattern that has been observed for adult *M. soledadinus* sampled along the altitudinal transects.

The separation of the *M. soledadinus*’ groups sampled along the two altitudinal transects was generally similar. First, carabid beetles sampled at the coast differed from the other sampled insects. Second, beetles sampled at higher elevation (200 m a.s.l. – St. Malo only - and 250 m a.s.l.) were distinct. In adult *M. soledadinus* sampled at 200 and 250 m a.s.l., the concentrations of free amino acids, and also intermediate acidic metabolites and polyols were increased as compared with carabid beetles from lowland habitats (50 m a.s.l.). These physiological responses have been reported previously in a large range of insect species subjected to cold temperatures^51,52^, including adults of *M. soledadinus*^21^. Higher levels of free amino acids are often considered as a reliable marker of thermal stress in insects, thus supporting the hypothesis of less favourable thermal conditions for adult *M. soledadinus* thriving at 200–250 m a.s.l at the Kerguelen Islands. Interestingly, we also found that the ranking of the insects from the two sampling sites, St. Malo and Molloy, was similar in the two altitudinal gradients, with a gradient of lower to higher altitudinal sampling sites along the first axis. It is very likely that decreasing temperatures along the two altitudinal transects represent a significant force shaping in a similar manner the physiology of adult *M. soledadinus*. At intermediate elevations, metabolic responses of the insects exhibited important inter-site variations, most probably resulting from the specific micro-environmental characteristics of the habitats and from inter-individual variation (age, sex, reproductive status, nutritional status at the time of collection). Of note, an individual collected at a given sampling point may have not spent its entire life at this point and may have moved to lower or higher elevation after its emergence (although larvae are likely to have much lower dispersal abilities compared with adults). Although this phenomenon would act to blur some of the interpretations made, our data suggest that its occurrence is not sufficiently frequent to override all patterns along transects.

## Conclusions

Recent climatic changes in the Kerguelen archipelago have positively influenced the invasion process of *M. soledadinus* both at lower altitude locations where populations of this species were largely absent before 2003, and at higher altitudes. Taken together, our data suggest that specimens collected along altitudinal transects are characterized by distinct physiological condition as compared with their lowland counterparts, supporting the idea of the colonisation of suboptimal highland habitats becoming gradually more suitable. Yet, thermal conditions remain suboptimal, and *M. soledadinus* sampled at higher altitudes most probably continue to experience significant temperature-imposed resource and physiological limitations, as revealed for instance by the accumulation of cold-stress molecular markers. Our data thus suggest that, if current temperature increase trends continue, this insect will likely flourish at its currently colonised altitudinal limits, and could even progress and colonise higher altitudes (given the availability of suitable habitat and trophic resources), while also extending its geographical distribution westwards, towards the currently colder, wetter and ice-capped part of the archipelago.

## Materials and Methods

### Insect sampling along environmental gradients

Adult *M. soledadinus* were hand-collected under stones in March 2013 at the Kerguelen Islands at three distinct localities: Molloy, St. Malo and Anse des Papous (Supplementary Fig. S1 and Table S4). At Molloy and St. Malo, two altitudinal transects were sampled, starting from the foreshore (0 m a.s.l.) and continuing up to 250 m a.s.l.; in both locations, beetles were sampled at 50 m elevational intervals along the transect (N = 6 sampling sites for each transect). At Anse des Papous, a horizontal low altitude transect was completed away from the seashore. This transect included eight sampling sites (0, 50, 100, 150, 200, 250, 300 and 400 m inland). At each sampling site, GPS coordinates were recorded, and a total of 86 adult *M. soledadinus* were collected: 50 individuals were sampled for morphometric and colorimetric assays (total protein and glycogen), 18 individuals (six replicates of three beetles) were collected for triglyceride assays, and 18 individuals (six replicates of three beetles) for GC-MS analyses. Upon collection, insects were directly plunged into microtubes containing 1 mL of 96% ethanol, and stored at −20 °C before further analyses. The physiology of insects may vary on a daily basis, depending on field conditions (e.g. temperature, relative humidity) or trophic status. As a result, particular attention was given to the meteorological conditions, and insect sampling was only carried out on days with similar weather (no rain, wind below 25 km/h, air temperature at the research station between 9 and 12 °C). Finally, insects were collected in the morning of three consecutive days to average environmentally-induced noise and limit possible effects of any circadian rhythm on the physiology of the insects.

### Morphological measurements

**Figure 7:**
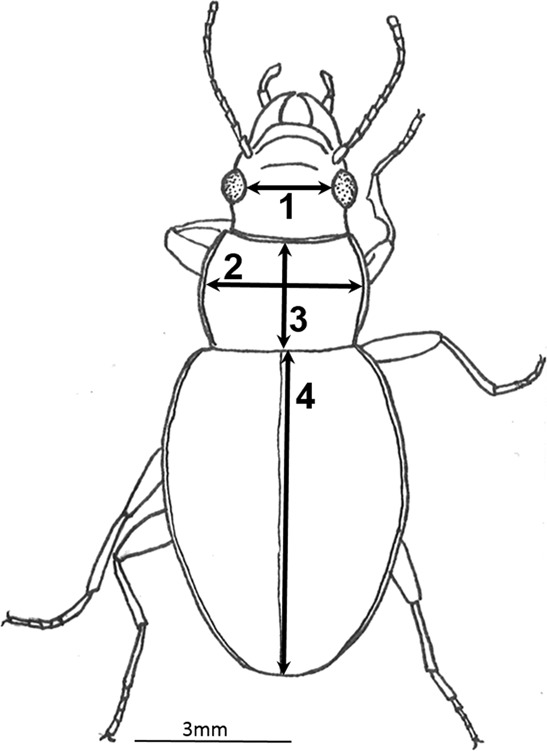
Morphological traits measured on adults of *M. soledadinus*: interocular width (1), pronotum width (2) and length (3) and right elytra length (4). This figure is an original drawing from T. Ouisse.

At each sampling site, 25 individuals (from the 50 sampled beetles) were randomly selected, except for specimens sampled at St. Malo at 0, 50 and 250 m a.s.l. for which only 12, 21 and 6 beetles were available, respectively. Sex of each beetle was determined under a stereo microscope. As often observed for the species at the Kerguelen Islands, sex ratio was biased toward males in 75% of the samples, and it was necessary to include additional females for the Anse des Papous transect (0, 100, 150, 250 and 300 m sampling sites). Pictures of each beetle were taken with a video camera (AxioCam ERc 5 s, ZEISS, Germany) connected to a stereo microscope. Inter-ocular distance, width and length of the thorax, length of the right elytra (Fig. 7) were measured by vectorial layouts with AxioVison software (repeatability was verified using a set of 20 individuals measured twice for each morphological trait: the mean proportion difference was 0.9, 2.6, 2.4 and 2.9 for inter-ocular distance, width and length of the thorax and right elytra length, respectively. Beetles were subsequently vacuum-dried for 15 h (Speed Vac Concentrator, MiVac, GENEVAC LTD, Ipswich, United Kingdom), before being individually weighed (Balance XP2U METTLER TOLEDO, Columbus, OH, d = 0.1 µg).

### Protein, glycogen and triglyceride contents

Body composition was determined from insects from the six sampling sites (0, 50, 100, 150, 200 and 250 m a.s.l.) from Molloy and St. Malo (altitudinal transects). For Anse des Papous (horizontal transect), body composition was determined from insects from five sampling sites (0, 100, 200, 300, and 400 m).

Protein and glycogen assays were performed following the protocol developed by Foray *et al*.^53^, which allows measurements of total protein and glycogen contents from the same sample. These analyses were conducted on the individuals that were used for morphometric assays. To have comparable sample numbers, six males and six females whose dry mass was distributed between the first and the third quartiles of the sample dry mass distribution were processed for each sampling site. Because of the biased sex ratio toward males in the samples, only three sampling sites of the St. Malo transect contained enough females to perform the assays (100, 150 and 200 m). Samples were homogenised in 180 µL of phosphate buffer for 90 s at 25 Hz using a bead-beating device (RETSCH MM301, RETSCH GBMH, Haan, Germany). Following low-spin centrifugation (180 *g*, 4 °C), 10 µL of the supernatant was used to measure body protein content using Bradford's technique, as described in Foray *et al*.^53^. Absorbance of the samples was read at 595 nm. The quantity of protein was calculated from a bovine serum albumin (CAS number: 9048–46–8) calibration curve. The remaining 170 µL of each sample was mixed with 20 µL of a solution of 20% Na_2_SO_4_ and 1200 µL of methanol-chloroform [ratio 2:1, volume:volume]. Samples were centrifuged (180 *g*, 4 °C) to precipitate glycogen into the pellet. Glycogen concentration was measured through a reaction with 70% anthrone (CAS number: 90-44-8), and the absorbance was read at 625 nm. Glycogen concentration in the samples was calculated based on a glucose standard calibration curve.

For the triglyceride assays, samples consisted of three hand-collected individuals. Six replicates were processed for the six sampling sites of Molloy and St. Malo (except for altitudes 0 and 150 m a.s.l. at St. Malo, for which only five replicates were available), and for five sampling sites of Anse des Papous (0, 100, 200, 300 and 400 m). We used the analytical procedure described in Laparie *et al*.^54^. Briefly, samples were vacuum-dried and weighed before being homogenised in 1050 µL of methanol-chloroform [ratio 1:2, volume:volume] for 90 s and 25 Hz using a bead-beating device. After centrifuging, a volume of 600 µL of the lower phase (chloroform + lipids) was transferred to clean microtubes and dried overnight in a fume cupboard. The lipid droplet was re-dissolved into 200 µL of Triton-BSA buffer and incubated for 10 min at 60 °C immediately before the assays. The manufacturer's instructions were followed for the triglyceride colorimetric assays (Triglycerides, kit reference CC02200, LTA SRL, Italy).

### GC-MS analyses: sample preparation and derivatisation

Environmental metabolomics has proven a powerful tool in the determination of insect-environment interactions (see reviews by^55,56^). Field-sampled organisms are expected to show high inter-individual variability, which we attempted to reduce by working on pools of three insects. Concentrations of non-structural carbohydrates, polyols, amino and organic acids were measured using a GC-MS platform as described in Khodayari *et al*.^57^. Analyses were carried out on pools of three hand-collected carabid beetles (N = 6 replicates for Molloy and St. Malo, and N = 5 replicates for Anse des Papous) to describe the metabolic phenotypes of adults from 17 sampling sites. Dried insect samples were re-dissolved in 600 µL of methanol-chloroform (ratio 2:1, volume:volume) and homogenised using a bead-beating device (RETSCH MM301, RETSCH GBMH, Haan, Germany) at 25 Hz for 90 s. A volume of 400 µL of ultrapure water was added to the samples that were then further centrifuged for 10 min at 4000 *g* and 4 °C. Then 90 µL of the supernatant, which contained polar metabolites, was transferred to new microtubes, and these aliquots were vacuum-dried (Speed Vac Concentrator, MiVac, GENEVAC LTD, Ipswich, United Kingdom). Dried samples were re-suspended in 30 µL of 20 mg mL^−1^ methoxyamine-hydrochloride (CAS Number: 593-56-6, SIGMA-ALDRICH, St Louis, MO, USA) in pyridine prior to incubation under orbital shaking at 40 °C for 60 min. Following incubation, 30 µL of N-methyl-bis(trifluoroacetamine) (BSTFA, CAS Number: 685-27-8) was added, and derivatisation was conducted at 40 °C for 60 min under agitation. Then, 1 µL of each sample was injected using the split mode (25:1). Calibration curves were run for 62 pure reference compounds at concentrations of 1, 2, 5, 10, 20, 50, 100, 200, 500, 750, 1000 and 1500 µM.

### Statistical analyses

For the three transects, samples collected from the foreshore are referred to henceforth as the ‘initial sampling point’. For each transect, the Euclidian distances between the initial sampling point and all other sampling points were calculated from the georeferenced coordinates. In order to investigate the effect of resource rarefaction with progression inland from the seashore, variables measured in individuals sampled from 0 to 200 m (coast proximity) were compared with measurements in individuals sampled beyond 200 m inland. This threshold was selected because it corresponds to the limit of seashore influence in similar environments^58^, which was corroborated by NDVI (Normalised Difference Vegetation Index) data from the Kerguelen archipelago, that decrease from the shore to 200 m inland, and stabilise further inland^59^. Site effect was not directly included as an explanatory variable because of its correlation with altitudinal variations; given the clustered coordinates along the coast, longitudinal effect was used as a proxy for inter-site differences.

Linear models were used to assess the effect of altitude, coast proximity, sex (if available) and z-scaled spatial coordinates on each of our measured variables (body size, protein, glycogen and triglyceride amounts), including the interactions between sex and the other variables, as well as between coast proximity and longitude (inter-site variations of coast proximity effect were considered). Interpretations of interactions were carried out using the *visreg* package^60^ and non-significant interactions were dropped. All morphological measurements were computed so that we obtained a general measure of body size. This computed body size value corresponds to the coordinates of the projection of the morphological measurements of each individual on the first axis of a Principal Component Analysis (*FactoMineR* package^61^), which accounted for 67.4% of the total variance.

Forty-six metabolic compounds were identified *via* the GC-MS procedure. Concentrations were homogenised (nmol.mg^−1^ of individual dry mass) and four molecules (cadaverine, ornithine, quinate, and ribose) showing extreme variations of concentrations in various samples were removed from subsequent analyses. First, we tested the effect of altitude, coast proximity and z-scaled spatial coordinates on individual metabolite concentrations. MANOVAs were performed to test for potential differences among experimental groups. Second, total metabolite amounts were summed for four chemical families (free amino acids, N = 16; organic acids, N = 8; polyols, N = 8; and sugars + phosphorylated sugars, N = 5; Supplementary Table S3) for each sampling site. Linear models were then used to compare the amounts of each metabolite family according to altitude, coast proximity and z-scaled spatial coordinates. Third, the concentration of each metabolite was log-transformed to normalise the distribution of model residuals. Linear discriminant analyses (LDA, *MASS* package^62^) were then run on individual metabolites sharing less than 80% correlation (N = 27 metabolites). Statistical significance of discrimination was confirmed using the Monte-Carlo permutation test (*P* < 0.001; 10,000 permutations). In a first LDA, we plotted the metabolic phenotypes of adult *M. soledadinus* from the three transects. This LDA revealed that our dataset of 27 metabolic compounds was better discriminated transect by transect, with 52.1% of the total variance being explained by the first axis (the between-group inertia being 10.7 times higher than the within-group inertia), and 47.8% by the second axis (Supplementary Fig. S2). LDA analyses were then performed for each transect. The distribution of model residuals was checked using QQ plots and Shapiro-Wilk tests for multivariate normality.

All analyses were carried out with R 3.3.0 statistical software^63^.

## Supplementary information

Supplementary Materials.

Supplementary Materials 2.

Supplementary Materials 3.

Supplementary Materials 4.

Supplementary Materials 5.

Supplementary Materials 6.

## Data Availability

The datasets generated during and/or analysed during the current study are available from the corresponding author on reasonable request.
