# Supplementary figures and images for: Effects of elevational range shift on the morphology and physiology of a carabid beetle invading the sub-Antarctic Kerguelen Islands
Source: Sci Rep. 2020 Jan 27;10:1234. doi: 10.1038/s41598-020-57868-0 (PMC6985133; doi:10.1038/s41598-020-57868-0)

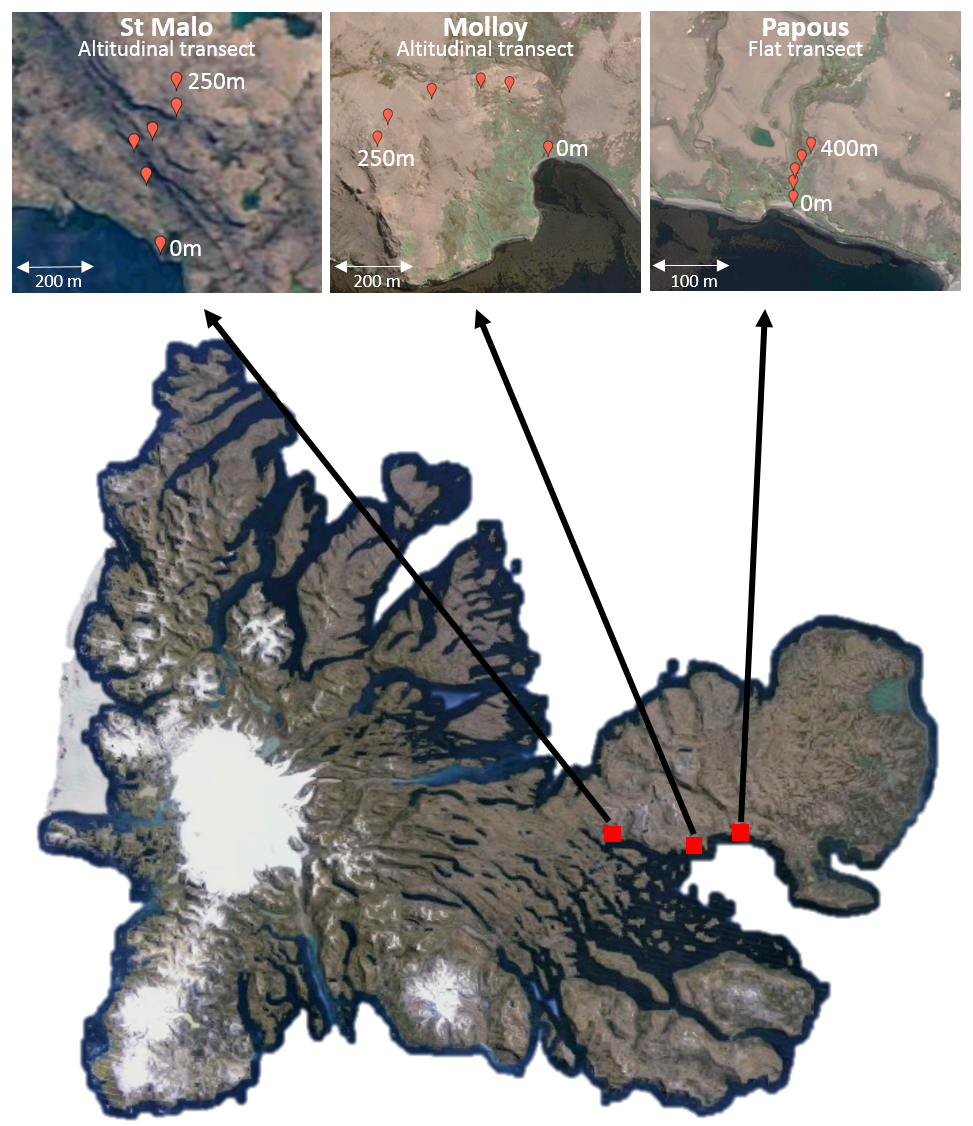

Supplement: Supplementary file 1 — Supplementary Materials. [file 41598_2020_57868_MOESM1_ESM.png]

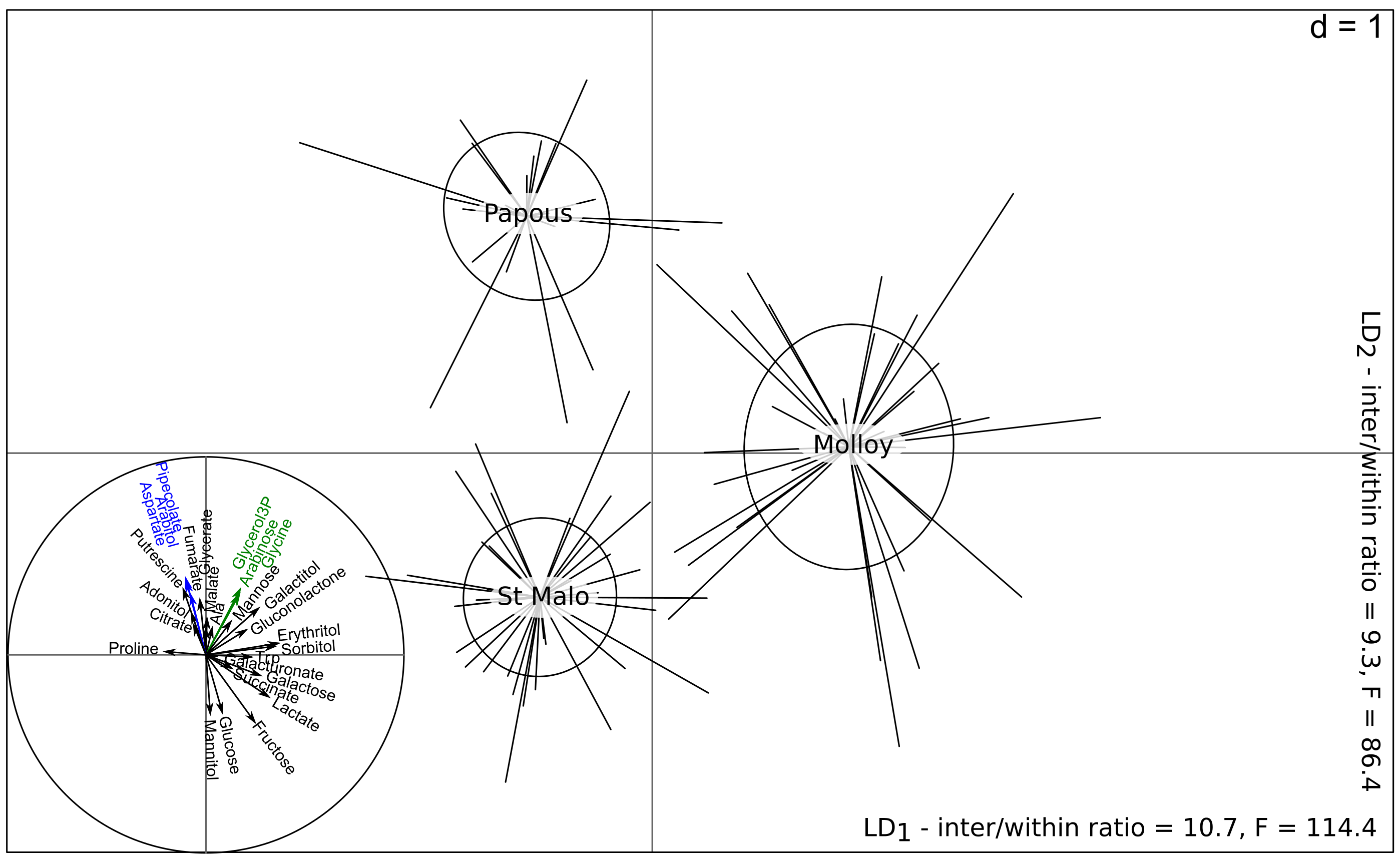

Supplement: Supplementary file 2 — Supplementary Materials 2. [file 41598_2020_57868_MOESM2_ESM.png]
