# Supplementary material for: Effects of elevational range shift on the morphology and physiology of a carabid beetle invading the sub-Antarctic Kerguelen Islands
Source: Sci Rep. 2020 Jan 27;10:1234. doi: 10.1038/s41598-020-57868-0 (PMC6985133; doi:10.1038/s41598-020-57868-0)
Supplement: Supplementary file 5 — Supplementary Materials 5. [file 41598_2020_57868_MOESM5_ESM.docx]

|  |  | **Altitude** | | **Sea proximity** | | **Longitude** | | **Sea proximity x longitude** | |
| --- | --- | --- | --- | --- | --- | --- | --- | --- | --- |
| **Compound** | **Metabolic Family** | F-value | *P* | F-value | *P* | F-value | *P* | F-value | *P* |
| Adonitol | Polyols | 0.0235 | 0.878438 | **9.7257** | **0.002472 **** | 1.4355 | 0.234158 | **7.2194** | **0.008656 **** |
| Alanine | Free Amino Acids | 0.6608 | 0.4185 | **16.8923** | **9.00E-05 ***** | 2.3031 | 0.1328 | 0.3841 | 0.5371 |
| Arabinose | Sugars | 0.3806 | 0.538921 | **6.7109** | **0.011254 *** | **8.8783** | **0.003751 **** | 1.0789 | 0.301848 |
| Arabitol | Polyols | 1.5442 | 0.21737 | 2.9106 | 0.09161 | 1.0156 | 0.31639 | 1.4875 | 0.22594 |
| Asparagine | Free Amino Acids | 2.6048 | 0.1102032 | **13.2486** | **0.0004649 ***** | **5.8606** | **0.0175862 *** | **6.259** | **0.0142497 *** |
| Aspartate | Free Amino Acids | 1.9869 | 0.16227 | **8.0718** | **0.005612 **** | **4.6173** | **0.034462 *** | 3.6914 | 0.058007 |
| Citrate | Organic Acids | **6.834** | **0.01056 *** | 1.9922 | 0.16172 | 3.0878 | 0.08244 | 0.7333 | 0.39421 |
| Citrulline |  | 0.7435 | 0.390928 | **10.7527** | **0.001504 **** | **5.7524** | **0.018628 *** | **4.5812** | **0.035156 *** |
| Erythritol | Polyols | 1.1252 | 0.29177 | 0.9309 | 0.33735 | 3.6875 | 0.05814 | 0.2205 | 0.63984 |
| Ethanolamine |  | 0.6366 | 0.42716 | **6.0092** | **0.01625 *** | **5.5255** | **0.02103 *** | 2.2546 | 0.13688 |
| Fructose | Sugars | 1.3231 | 0.25323 | **4.2274** | **0.04281 *** | 0.0112 | 0.91606 | 0.016 | 0.89977 |
| Fumarate | Organic Acids | 3.5124 | 0.064306 | **10.8623** | **0.001427 **** | **6.6526** | **0.011601 *** | 1.9077 | 0.170794 |
| GABA | Free Amino Acids | 0.0001 | 0.991255 | **9.9973** | **0.002166 **** | **10.6273** | **0.001597 **** | 1.2865 | 0.259854 |
| Galactitol | Polyols | 0.0279 | 0.867805 | 0.1321 | 0.717207 | **7.3697** | **0.008015 **** | 0.6597 | 0.418911 |
| Galactose | Sugars | 0.1251 | 0.7244 | 0.9525 | 0.3318 | 1.1909 | 0.2782 | 2.323 | 0.1311 |
| Galacturonate | Organic Acids | **4.9153** | **0.02926 *** | 1.651 | 0.20227 | 0.5026 | 0.48029 | 0.6609 | 0.41847 |
| Gluconolactone |  | 1.2741 | 0.26214 | 3.3622 | 0.07017 | **4.4151** | **0.03855 *** | 2.0931 | 0.1516 |
| Glucose | Sugars | 3.3526 | 0.07056 | 1.2721 | 0.2625 | 1.1622 | 0.28402 | 2.0218 | 0.15867 |
| Glutamate | Free Amino Acids | 0.7222 | 0.39779 | **17.7079** | **6.30E-05 ***** | **7.7708** | **0.006534 **** | **6.378** | **0.013388 *** |
| Glycerate | Organic Acids | 2.0177 | 0.1591 | **16.8068** | **9.35E-05 ***** | 2.3981 | 0.1252 | 0.4704 | 0.4947 |
| Glycerol3P | Polyols | 0.2412 | 0.624603 | **6.8406** | **0.010522 *** | **10.6085** | **0.001612 **** | 0.0607 | 0.805906 |
| Glycine | Free Amino Acids | 0.3823 | 0.538025 | 2.1491 | 0.146298 | **8.332** | **0.004924 **** | 0.145 | 0.704267 |
| Inositol | Polyols | 3.2391 | 0.07541 | **3.9634** | **0.04967 *** | 2.3717 | 0.12723 | 0.0057 | 0.93999 |
| Isoleucine | Free Amino Acids | 0.6084 | 0.43754 | **8.911** | **0.00369 **** | **5.9764** | **0.01654 *** | **4.3574** | **0.03981 *** |
| Lactate | Organic Acids | **8.8848** | **0.003739 **** | 2.2634 | 0.136131 | 1.0031 | 0.319371 | 0.0433 | 0.835724 |
| Leucine | Free Amino Acids | 2.5512 | 0.1138791 | **11.6776** | **0.0009683 ***** | **6.3664** | **0.0134691 *** | **6.6516** | **0.0116066 *** |
| Lysine | Free Amino Acids | 1.613 | 0.2075 | **10.1409** | **0.00202 **** | **4.411** | **0.03864 *** | **4.6039** | **0.03472 *** |
| Malate | Organic Acids | 0.3601 | 0.55 | 0.1369 | 0.7123 | 0.9635 | 0.3291 | 0.9645 | 0.3288 |
| Mannitol | Polyols | 3.2094 | 0.07673 | 2.2482 | 0.13743 | 0.9414 | 0.33464 | 0.5238 | 0.47118 |
| Mannose | Sugars | 0.6 | 0.44069 | 1.7401 | 0.19063 | 3.6174 | 0.06053 | 0.7337 | 0.39407 |
| Methionine | Free Amino Acids | 1.4699 | 0.2286756 | **12.6246** | **0.0006209 ***** | **7.6435** | **0.0069705 **** | **5.6561** | **0.0196101 *** |
| Phenylalanine | Free Amino Acids | 1.4118 | 0.238021 | **10.3032** | **0.001867 **** | **5.6484** | **0.01969 *** | **4.1213** | **0.045437 *** |
| Free Phosphate |  | 0.6753 | 0.41348 | **29.991** | **4.24E-07 ***** | **8.0934** | **0.005551 **** | **4.5818** | **0.035144 *** |
| Pipecolate | Organic Acids | 1.1465 | 0.2872883 | **15.4845** | **0.0001682 ***** | **7.9109** | **0.0060868 **** | 3.5896 | 0.0615015 |
| Proline | Free Amino Acids | 3.0915 | 0.082262 | 3.0493 | 0.084344 | 0.0412 | 0.839571 | **8.4994** | **0.004529 **** |
| Putrescine |  | 0.8265 | 0.36582 | **5.0816** | **0.02672 *** | 3.249 | 0.07497 | **5.6422** | **0.01976 *** |
| Serine | Free Amino Acids | 2.4874 | 0.118434 | **10.8026** | **0.001469 **** | 2.9654 | 0.088658 | **6.3828** | **0.013354 *** |
| Sorbitol | Polyols | 3.0449 | 0.08456 | 0.0513 | 0.82133 | 3.6666 | 0.05884 | 0.927 | 0.33833 |
| Succinate | Organic Acids | 1.0941 | 0.2985 | 0.1473 | 0.7021 | 0.4268 | 0.5153 | 0.003 | 0.9562 |
| Threonine | Free Amino Acids | 1.3869 | 0.242171 | **10.5906** | **0.001626 **** | **4.9475** | **0.028747 *** | **5.4043** | **0.022444 *** |
| Tryptophan | Free Amino Acids | 2.9885 | 0.08744 | **5.3553** | **0.02304 *** | 1.7683 | 0.1871 | 0.014 | 0.90598 |
| Valine | Free Amino Acids | 0.2794 | 0.598459 | **10.26** | **0.001907 **** | **6.2222** | **0.014528 *** | 3.0464 | 0.08449 |

**Supplementary Table S3** Numerical outputs of the MANOVA computed on the 42 quantified metabolites, investigating the effects of altitude, coast proximity and longitude on metabolite concentrations (significant relationships are highlighted in bold).
