# Supplementary material for: Effects of elevational range shift on the morphology and physiology of a carabid beetle invading the sub-Antarctic Kerguelen Islands
Source: Sci Rep. 2020 Jan 27;10:1234. doi: 10.1038/s41598-020-57868-0 (PMC6985133; doi:10.1038/s41598-020-57868-0)
Supplement: Supplementary file 6 — Supplementary Materials 6. [file 41598_2020_57868_MOESM6_ESM.docx]

**Supplementary Table S4** GPS localisation, altitude and distance from the initial point of the sampling sites along each of the three transects.

| **Sampling Site** | **Latitude** | **Longitude** | **Altitude (m)** | **Distance from initial sampling point (m)** |
| --- | --- | --- | --- | --- |
| Papous 0 | -49.345091 | 70.170657 | 0 | 0 |
| Papous 50 | -49.344643 | 70.170674 | 0 | 50 |
| Papous 100 | -49.344125 | 70.170649 | 0 | 107 |
| Papous 150 | -49.343732 | 70.170666 | 0 | 151 |
| Papous 200 | -49.343355 | 70.170777 | 0 | 193 |
| Papous 250 | -49.342906 | 70.171034 | 0 | 244 |
| Papous 300 | -49.342513 | 70.171451 | 0 | 292 |
| Papous 400 | -49.341733 | 70.172401 | 0 | 394 |
| Molloy 0 | -49.340408 | 69.82201 | 0 | 0 |
| Molloy 50 | -49.336666 | 69.820862 | 50 | 427 |
| Molloy 100 | -49.33484 | 69.81988 | 100 | 646 |
| Molloy 150 | -49.334229 | 69.821391 | 150 | 705 |
| Molloy 200 | -49.332949 | 69.823367 | 200 | 859 |
| Molloy 250 | -49.331579 | 69.823382 | 250 | 1018 |
| St Malo 0 | -49.361968 | 70.06634 | 0 | 0 |
| St Malo 50 | -49.357872 | 70.062506 | 50 | 535 |
| St Malo 100 | -49.357642 | 70.059627 | 100 | 691 |
| St Malo 150 | -49.358327 | 70.054793 | 150 | 941 |
| St Malo 200 | -49.359952 | 70.050551 | 200 | 1182 |
| St Malo 250 | -49.361342 | 70.049556 | 250 | 1242 |
